# Stress, dyadic coping, and relationship satisfaction: A longitudinal study disentangling timely stable from yearly fluctuations

**DOI:** 10.1371/journal.pone.0231133

**Published:** 2020-04-09

**Authors:** Petruta P. Rusu, Fridtjof W. Nussbeck, Lorena Leuchtmann, Guy Bodenmann

**Affiliations:** 1 Department of Educational Sciences, University “Stefan cel Mare” of Suceava, Suceava, Romania; 2 Department of Psychology, University of Konstanz, Konstanz, Germany; 3 Department of Psychology, University of Zurich, Zurich, Switzerland; University of California Los Angeles, UNITED STATES

## Abstract

The aims of the present study are to analyze the associations of different forms of dyadic coping (i.e., own supportive dyadic coping = OSDC; perceived supportive dyadic coping provided by the partner = PSDC; common dyadic coping = CDC) with relationship satisfaction, and to investigate whether these effects differ depending on the amount of perceived stress. In 240 couples, the different forms of dyadic coping and stress of both partners were assessed annually across 5 measurement points. Data was analyzed by dyadic multilevel models, which allow for disentangling between-person (overall, timely stable) from within-person (yearly, time specific) variations. The results revealed that all different forms of dyadic coping enhanced overall and yearly relationship satisfaction. At the same time, relationship satisfaction depends on the amount of overall and yearly stress. Interestingly, for PSDC, we found that the more a member of the couple was supported by the partner yearly (time-specific PSDC) and the more the member was stressed overall (timely stable), the higher the member scored on relationship satisfaction. For CDC, we found that yearly CDC beyond the overall level of CDC interacted with the timely stable amount of stress. Dealing together with stress and perceiving the partner as helpful were especially beneficial for relationship satisfaction. Findings highlight the importance of addressing specific forms of dyadic coping in intervention and prevention programs for couples.

## Introduction

Dyadic coping refers to the stress management process in the context of romantic relationships [1,2] and has repeatedly been found to be linked with relationship functioning and individual psychological and physical well-being of the partners [3–5]. Several forms of dyadic coping can be distinguished, and the results of a meta-analysis suggest that they differ regarding their importance for relationship satisfaction with supportive dyadic coping (supportive reactions on the other partner's stress signals) and common dyadic coping (joint efforts of a couple to cope with adversities) being the most important dimensions [4]. In the context of clinical samples (i.e., couples suffering from illness), common dyadic coping appeared to be particularly important with a stronger impact on long-term relationship-specific outcomes than other forms of dyadic coping [6]. Although, the beneficial effects of support have been documented in both adverse, highly stressful contexts (e.g., couples coping with illness) and less stressful contexts (e.g., positive challenges, such as support provided to the partners in pursuing their own goals; see for review Feeney and Collins [7]), there is limited research on community samples comparing the effects of different forms of dyadic coping on relationship satisfaction (e.g., comparing supportive and common dyadic coping). Moreover, to date, most studies tended to focus on general associations of dyadic coping with marital outcomes, but without disentangling timely stable from situation-specific fluctuations in these associations. Given that some forms of dyadic coping might be particularly important for relationship satisfaction only in moments when one partner is highly stressed, distinguishing between overall and yearly dyadic coping and stress in a longitudinal study would help us to understand better what kind of dyadic coping is beneficial related to the level of stress and for whom. The aims of the present study are the following: (a) to disentangle overall (timely stable, between-level) and yearly (time specific, within-level) effects of supportive dyadic coping and common dyadic coping on relationship satisfaction in a longitudinal study based on data from a community sample, (b) to compare the associations of overall and yearly supportive and common dyadic coping with relationship satisfaction, and (c) to analyze whether the amount of overall and yearly stress influences the association between the different forms of dyadic coping and relationship satisfaction by investigating between- and within-level stress as a moderator in these associations.

### Dyadic coping and relationship satisfaction: Between- and within-persons analysis

The Systemic Transactional Model (STM) [2] emphasizes the interdependence between partners’ stress (dyadic stress) and partners’ resources to cope with stress (dyadic coping). The cross-over of individual stress (such as work stress) from one partner to the other and partners’ stress from common sources (such as stress related to child caring) determine joint appraisal of stressful situations and trigger dyadic coping strategies in addition to individual coping. Strategies of dyadic coping are expressed in the form of supportive reactions on the other partner's stress (supportive dyadic coping), conjoint efforts to cope with stress (common dyadic coping) and delegating one’s responsibilities to the other partner (delegated dyadic coping). The present study focuses on supportive and common dyadic coping, as these two forms have been shown to be most relevant for relationship satisfaction [4]. Studies involving dyadic data analyses generally consider two types of dyads: distinguishable dyads (when the two members of the dyad can be distinguished, such as in heterosexual couples distinguished by gender) and indistinguishable dyads (when there is no variable to distinguish the two persons within the dyad, such as in homosexual couples) [8,9]. As we considered heterosexual couples, partners are distinguished by gender in our study, which is a within-dyads variable. Gender is also an important variable in order to investigate differences between women and men in terms of the associations of dyadic coping with relationship satisfaction and the moderating role of stress. One partner’s dyadic coping can have a negative effect on his/her own relationship satisfaction (actor effect), but also on his/her partner relationship satisfaction (partner effect).

A considerable amount of studies investigating dyadic coping and relationship outcomes have been published in the past two decades. Dyadic coping has been linked to higher levels of relationship satisfaction [4], relationship stability [10], and partners individual well-being [3,5,11] better psychological adjustment to chronic stress (e.g., medical illness) [12,13] and better physiological stress responses, such as cortisol recovery and increased immune reactivity [14–16].

Despite the importance of these studies in advancing the knowledge about dyadic coping, most of them focused on the between-person variations (i.e., timely stable differences) in linking dyadic coping with individual and relationship outcomes. Specifically, these studies showed that partners who report higher levels of dyadic coping on average experience higher levels of relationship quality and individual well-being than people who experience less dyadic coping in their relationship. Although, these findings contribute to a better understanding of couple interactions, it is also important to analyze time-specific (within-person) differences in order to understand fluctuations in relationship satisfaction (e.g., ups and downs across different time-units such as years, weeks, or days) based on dyadic coping. An analysis of dyadic coping at the within-level allows us to analyze whether partners report higher levels of relationship satisfaction in times when they experience more dyadic coping (e.g., received and provided partner support and common dyadic coping) than in other times. The few studies investigating the within-persons effects showed that daily spousal support was positively associated with relationship closeness, positive affect, and relationship satisfaction [17–19] and negatively associated with negative affect and emotional exhaustion [17,19,20]. Moreover, existing findings showed that women tend to be more affected by the momentary support received from their partners. For example, Hilpert and colleagues [18] found significant within-persons effects in the association of received supportive dyadic coping and relationship satisfaction only for women; namely, women, but not men reported higher levels of relationship satisfaction on days when they received higher levels of partner support. For a further investigation of the link between dyadic coping and relationship satisfaction, the present study investigates both the between- and within-persons effects in linking dyadic coping with relationship satisfaction longitudinally across four years.

### Supportive dyadic coping vs. common dyadic coping predicting relationship satisfaction in community and clinical samples

Supportive dyadic coping and common dyadic coping have both been shown to be positively associated with relationship functioning (for review see [4]). Supportive dyadic coping has repeatedly been linked to long-term relationship functioning and stability in community samples [4,10,21]. There is evidence that receiving dyadic coping from the partner is a stronger correlate of relationship satisfaction than the provision of dyadic coping by oneself [4,22]. However, the Conceptual Model for Thriving through Relationships [7] highlights that effective support involves both *receiving support* in an active and receptive manner (e.g., expressing needs, expressing gratitude) and *providing responsive support*. People have an inherent need of providing support to others and there is evidence linking providing support to positive psychological and physical outcomes for both provider and recipient [4,23–25]. Other studies indicated that providing support has been associated to emotional exhaustion for the provider, by simultaneously increasing the recipient's feelings of inefficacy and incompetence and by decreasing their self-esteem [26,27]. Moreover, unwanted support has been related to relationship conflict and negative emotions in couples coping with chronic illness of one partner [28]. Rafaeli and Gleason [29] described skilled support within couple relationships as a function of timing (*when* the support is provided), content (*what* type of support is needed, e.g., emotional or instrumental support), process (*how* the support is provided, e.g., invisible and nondirective support being more effective) and reciprocity (*who* is the support provider vs. receiver, equity between giving and receiving support being beneficial for both individuals and couples). The Social Support Effectiveness Model [30] posits that the support provided should match the needs of the recipients in terms of *quantity* (the amount of support provided must meet the support needed by recipient) and *quality* (the type of support provided, must meet the needs of recipient in terms of receiving emotional, instrumental, or informational support).

Considering the aforementioned models and findings, further studies are needed for clarifying the situations under which support is effective and beneficial for both recipients and providers. In order to answer these questions, the current study will address an important methodological issue by examining provided as well as received supportive dyadic coping and by disentangling between- from within-effects in the association of dyadic coping with relationship satisfaction. In contrast to existing studies, the present study combines timely stable and yearly variations of dyadic coping and stress in order to enhance our understanding of the stable and dynamic associations. Generalizing the findings of the cross-sectional studies and daily diary studies, we assume that women and men exposed to high levels of stress may benefit more from the support received from the partner than couples experiencing lower levels of stress at the between as well as at the within level.

With respect to common dyadic coping, it has been proven to be particularly important for clinical samples and has been related to positive outcomes for couples where one partner had a diagnosis of physical illness (diabetes, cancer) [6,12,22] or psychological disorder (depression, anxiety) [31]. However, only few studies compared the distinct effects of supportive and common dyadic coping. For example, Falconier and colleagues [4] did not find any significant differences between the effect of supportive dyadic coping and common dyadic coping on relationship satisfaction, although, descriptively the effect of common dyadic coping was stronger. However, 83% of considered studies in their meta-analysis were based on data from cross-sectional studies. In contrast, a longitudinal study investigating couples dealing with chronic illness (i.e., breast cancer) found that higher levels of common dyadic coping were related to increases in relationship quality for both partners whereas supportive dyadic coping did not have any effect [13].

In general, a predominant effect of common dyadic coping compared to supportive dyadic coping was found in studies focusing on couples coping with illness. Specifically, these studies showed that mutuality (empathizing with the partner) and we-ness (partners’ sense of unity and togetherness in couple) predicted an increase of effective coping and better adjustment to stress [32–35]. Thus, for couples facing chronic and uncontrollable stress (such as chronic illnesses), common dyadic coping efforts seem to be particularly valuable for relationship functioning. Moreover, existing studies suggest that common dyadic coping may be more beneficial for women’s coping with stress. For example, Falconier and colleagues [36] found that common dyadic coping moderated the negative association between women’s immigration stress and their own as well as their partners’ marital satisfaction. Similarly, Rottman and colleagues [13], in a study including women with breast cancer and their male partners, indicated that women’s perception of common dyadic coping was particularly related to both their own and their partners positive dyadic outcomes. These findings are consistent with the STM [2], where common dyadic coping is expected in situations when the stressor affects both partners similarly at the same time, thus, when the stress is a “we-stress”. Severe medical conditions represent a typical shared experience of demands, as both partners are affected by the illness in the sense of “we-disease” [37]. Therefore, common dyadic coping may be particularly important when a couple is confronted with chronic “we-stress” (i.e., high overall level of stress).

### Stress as a moderator in the association of dyadic coping and relationship satisfaction

According to the stress-cascade process within STM [2], partners experiencing low levels of stress external to their relationships (e.g., job stress) can cope with stress individually. However, when exposed to chronic and high levels of stress (from external sources or relationship related stress), more resources are needed and partners engage in dyadic coping in addition to individual coping, as they need to share the stress (appraising the stress as a “we-stress”), need to receive support and need to be involved in collaborative coping together with their partners. Moreover, some forms of dyadic coping may be particularly important for stress recovery and positive interactions between partners in highly stressful situations. For example, positive dyadic coping received from the partner was associated with physiological recovery from experimentally induced stress by using a public speaking task [15]. People exposed to high levels of stress, when the demands exceed their coping resources, may be more likely to benefit from receiving support from their partner and joint coping efforts than the ones who face lower levels of stress. Nevertheless, stress can directly impact dyadic coping in couples, as support providers may not be able to address their partner's needs and support recipients may not benefit equally from different dyadic coping strategies. Neff and Karney [38] suggest that stress negatively affects relationship satisfaction by a) creating additional relationship problems (e.g., couples experiencing financial strain may need to work more hours; parents of children with special needs need more time and energy for child care) and b) hindering partners energy and resources necessary to cope effectively with stress. Based on the Social Support Effectiveness Model [30], support effectiveness in stressful situations should match the needs of the recipient. Thus, highly stressful situations may evoke more needs for receiving supportive dyadic coping or for joint efforts to cope with stress, so that research studies examining dyadic coping and relationship outcomes should analyze the interaction between dyadic coping and stress in predicting relationship satisfaction. Taken together, the aforementioned theoretical models and empirical findings suggest that stress triggers dyadic coping (at micro-level, one partner reacts to the other partner’s stress) and dyadic coping is positively associated with relationship satisfaction (at macro-level, perceiving the partner as being supportive in stressful situations will lead to high levels of relationship satisfaction). At the same time, support in couple may be more important in certain life situations than in others (e.g., in highly stressful circumstances, dyadic coping may be a better predictor of relationship satisfaction than in less stressful circumstances). Therefore, dyadic coping may interact with the stress level in predicting long-term relationship satisfaction.

### The present study

Drawing from existing studies and based on the STM [2], the current study aimed to investigate the association of supportive and common dyadic coping with relationship satisfaction longitudinally and to analyze the moderating role of stress in this association. Specifically, we investigated the associations of each partner dyadic coping (OSDC, PSDC and CDC) with his/her own relationship satisfaction (actor effects) and his/her partner relationship satisfaction (partner effects). Moreover, we examined the associations between male and female stress with own relationship satisfaction (actor effect) and partner relationship satisfaction (partner effects) and how male and female stress moderated the actor and partner effects of dyadic coping on relationship satisfaction (OSDC, PSDC and CDC). First, we aimed to disentangle overall and yearly effects of supportive dyadic coping (provided to and received from the partner) and common dyadic coping on relationship satisfaction. Based on the findings of previous studies that investigated separately between-person (timely stable) and within-person (time-specific) variations, we predicted that both overall (timely stable) and yearly (time specific) supportive and common dyadic coping would be positively associated with relationship satisfaction. Second, we aimed to differentiate the effects of the three dyadic coping facets on relationship satisfaction. Only few previous studies considered both between- and within- persons associations of different facets of dyadic coping with relationship satisfaction. Therefore, comparing the timely stable and time-specific effects of the three facets of dyadic coping in the present study is an exploratory investigation and thus no a priori hypotheses were formulated regarding the magnitude of these effects. Third, according to the stress-cascade process within the STM [2] we sought to investigate stress as a moderator in the association of dyadic coping with relationship satisfaction. Specifically, we assumed that partners exposed to higher levels of stress would benefit more from supportive and common dyadic coping than the ones experiencing lower levels of stress. In addition, based on the Social Support Effectiveness Model [30] we predicted that receiving supportive dyadic coping and common dyadic coping are more beneficial to relationship satisfaction than providing supportive dyadic coping if the couple experiences higher between- and within levels of stress. Based on previous studies [13,18,36], we also predicted that supportive dyadic coping and common dyadic coping may be particularly important for women’s relationship satisfaction.

## Method

### Sample

The present study examined data from a large-scale study addressing different relationships variables. This data was used in several previous publications (see S1 Appendix). All relevant information related to the participants and procedure is repeated here. The present article is the only one that disentangles timely stable effects from yearly fluctuations of different forms of dyadic coping and stress on relationship satisfaction; thus, the current results do not overlap with previous publications. Couples were recruited by announcements in newspapers and on the radio and had to be in their current relationship for at least one year to be eligible. Three hundred sixty-eight heterosexual Swiss couples participated at the first measurement occasion. Couples were aware that they participated in a longitudinal study and were annually invited to the following occasions of measurement. To date, data from five occasions of measurement (four years) are available. At the first measurement occasion, couples were aged between 20 and 80 years (women: *M* = 47.2, *SD* = 18.3; men: *M* = 49.3, *SD* = 18.3) and were in their current relationship between 1 and 60 years (*M* = 21.2 years, *SD* = 18.1). Eighty-five percent of the partners lived together, 66% percent were married, and 65% had children. Participant's level of education and income suggests that it is a middle-class sample.

At time 1 (T1), 368 couples participated, at time 2 (T2) 302 couples, at time 3 (T3) 255 couples, at time 4 (T4) 228 couples, and at time 5 (T5) 227 couples. Dropout reasons were separation/divorce (41 couples), widowhood (7 couples), not wanting to or not being able to participate anymore (95 couples), or unknown reasons (18 couples). Additionally, 27 couples paused at time 2 (i.e., did not participate at this measurement point but assured to participate at the following measurement points), 47 couples paused at T3, 51 couples paused at T4, and 30 couples paused at T5. Couples who dropped out had a lower education than couples that still participated at T5 (women: *U* = –2.79, *p* = .005; men: *U* = –2.11, *p* = .035) and women who still participated at T5 had a lower income at T1 (*U* = –2.34, *p* = .019). With regards to any other target variables, or other variables such as age or relationship duration, dropouts did not differ from couples that still participated at T5. In order to disentangle timely stable from situation-specific effects, we considered those couples for analyses that participated at least at measurement point 1 and 4 or 5 in order to be able to investigate committed relationships and to have enough longitudinal information. Couples considered in this study do not differ from those not considered in this study with respect to the study variables (MANOVA: *F*(7;359) = 1.949; *p* = .06). Running additional *t*-test between considered and not considered participants on all study variables at T1, revealed no but one statistically significant difference: women of couples who are not considered in the study report higher own supportive dyadic coping (0.167 scale points). Hence, the considered and not considered couples are highly comparable at least in the beginning of the study.

Since we consider couples providing scores at all measurement occasions but also those providing at least scores at T1 and T4 or T1 and T5, we investigated differences between couples who paused at a given point in time and those who provided data at the same point in time. Overall, Little’s MCAR test [39] revealed that the data are most probably not missing completely at random (χ^2^ = 1245.696, *df* = 635, *p* < .001). A further inspection of the rate of missing data revealed that for any bivariate association of our study variables at least 80% of the data are present. Furthermore, we ran *t*-tests on all study variables of preceding occasions of measurement between the group of couples who paused at a given point in time and those who did participate at that given point in time. Out of the 120 tests, only 16 were statistically significant (no Type-I error correction) and mixed in the direction of effects (sometimes couples who paused showed more favorable scores on preceding occasions of measurement sometime these scores were less favorable). The effects themselves were not very pronounced (maximally 0.30 scale points). Hence, we consider missing data to be missing at random allowing us to use random-effects modeling approaches. The study was approved by the Ethics Committee of the Faculty of Philosophy of University of Zurich (Switzerland), Approval Number: 17.8.2.

### Procedure

When couples came to the laboratory, they were advised about the procedure and provided written informed consent. They then completed questionnaires in separate rooms and attended three videotaped interaction tasks not relevant for the current research question. Partners were reimbursed with 100 CHF each (approximately 105 USD). Couples were invited to the laboratory again annually across the next 4 years (T2, T3, T4, T5) and the same procedure took place with increasing reimbursement each year (10 CHF = approximately 11 USD).

### Measures

#### Dyadic coping

Dyadic coping was measured using the German version of the Dyadic Coping Inventory (DCI; [40]). The DCI assesses different forms of dyadic coping (e.g., common dyadic coping, supportive dyadic coping, delegated dyadic coping) as perceived by oneself and as perceived by one's partner. In the current study, we used the subscales measuring common dyadic coping (CDC), supportive dyadic coping provided by oneself (OSDC = own supportive dyadic coping; self-report), and supportive dyadic coping provided by one's partner (PSDC = perceived supportive dyadic coping provided by the partner; partner report). CDC, OSDC, and PSDC are measured by 5 items each (e.g., CDC: “We help one another to put the problem in perspective and see it in a new light”; OSDC: “I show empathy and understanding to my partner”; PSDC: “My partner shows empathy and understanding to me”). All items were rated on a 5-point frequency scale (1 = *very rarely*, 5 = *very often*). Various studies across different cultures have demonstrated high reliability and good validity. In the current study, internal consistencies for men and women at all five measurement points were acceptable, ranging from Cronbach's α = .67 to α = .87.

#### Stress

Stress was measured using the German Multidimensional Stress Questionnaire (MSQ-P) [41,42]. Both partners rated how stressful they perceived eight different areas of their life (job/education, social contacts, leisure time, children, family of origin, habitation, finances, daily hassles) within the last twelve months on a 4-point scale ranging from *not at all* to *very*. Given that higher perceived stress in one area of life does not need to go along with higher perceived stress in another area of life, we did not calculate the internal consistency of the scale.

#### Relationship satisfaction

Relationship satisfaction was measured using the German version of the Relationship Assessment Scale (RAS; [43,44]. The seven items were rated on a 5-point scale (various anchors depending on the content of the items; e.g., "How often do you wish you had not gotten into this relationship?" (reversely coded)). Internal consistencies for men and women at all five-measurement points were acceptable (Cronbach's α ranging from α = .83 to α = .89).

### Analytic strategy

**Fig 1:**
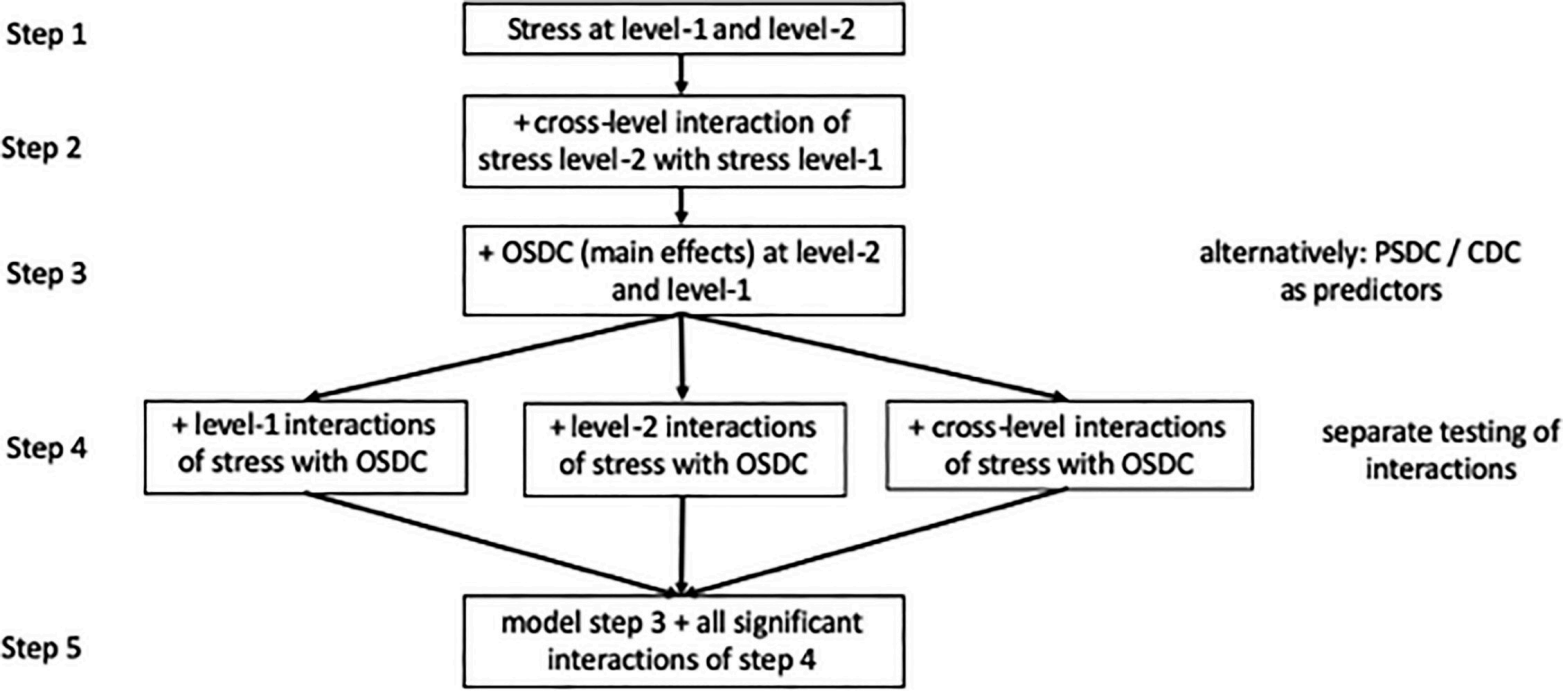
Flow chart of the stepwise analysis procedure for research question 3 with Own Supportive Dyadic Coping (OSDC) as exemplary independent variable. Step 1: determining the necessary random effects structure with stress as predictor at level-1 and level-2. Step 2: Additionally, integrating cross-level interactions of stress. Step 3: Entering main effects of OSDC at level-1 and level-2. Step 4: incorporating interactions of stress with OSDC separately for level-1 interactions, level-2 interactions, cross-level interactions due to the complexity of the models. Step 5: model with stress at level-1 and level-2, cross-level interaction of stress, main effects of OSDC, and all interaction effects of step 4 that turned significant when entered separately.

In order to examine our research questions, we ran longitudinal random effects models for dyadic data with within-person variability for female and male partners at level-1 and between-couples variability across male partners and female partners at level-2 [45–47]. These models treat the three-level data structure (time within partners within couples) as a two-level model with correlations of random effects at level-2 reflecting the dependency due to couple membership. In the estimated models answering research questions 1 and 2, dyadic coping competencies of both partners were entered as person-mean centered predictors at level-1 (effects indicated by *β*) and grand-mean centered predictors at level-2 (effects indicated by *γ*). In all tables presenting results, *a* (actor-effect) indicates a predictor measured for one (male or female) partner and predicting his/her own relationship satisfaction; *p* (partner-effect) indicates a predictor measured for one partner and predicting the other partner’s relationship satisfaction. Level-1 effects depict the impact of year-specific dyadic coping competencies on relationship satisfaction for that year; level-2 effects depict the impact of overall dyadic coping competencies on the average relationship satisfaction across the years. For both (male and female) partners, there are (level-specific) actor effects depicting the impact of one's own dyadic coping competencies on one's own relationship satisfaction and partner effects depicting the impact of the partner's dyadic coping competencies on one's own relationship satisfaction. Due to the complexity of the models, we first ran models with one dyadic coping competency at a time as predictor in order to shed light on the association of the dyadic coping facet with relationship satisfaction. We then estimated a model with all dyadic coping facets simultaneously to investigate the differential impact of the dyadic coping facets on relationship satisfaction. Finally, we added stress as a predictor at both levels. As these models are extremely complex, we opted for a stepwise modeling strategy (see Fig 1) to identify all necessary effects. In step 1, we modeled the impact of stress on relationship satisfaction at level-1 (yearly level) and -2 (overall level). In step 2, cross-level interactions were added to the model depicting if the impact of yearly stress (level-1) changes for different levels of overall stress (level-2). In Step 3, one of the dyadic coping facets (OSDC, PSDC, and CDC, respectively) was entered as predictor at levels-1 and -2 into the model, depicting yearly-and overall influences. Step 4 consists of many "sub-steps" where we tested interactions of stress and the DC facets at level-1 (year-specific interactions), at level-2 (overall interactions), and cross-level interactions (changes in year-specific influences due to overall influences). In step 5, we considered the model of step 3 and added the significant effects of step 4 simultaneously.

Importantly, we chose a "symmetric" modeling strategy, that is, if an effect turned significant for male (female) partners it was also entered for female (male) partners according to the structure of Actor-Partner-Interdependence Models (APIM) [9]. For all models, we focused on fixed-effects estimates (the average estimates across all couples). Additionally, we allowed for random effects estimates to account for differential effects between couples, but these effects are less important for our research questions. All models have been specified using the LME4 package [48] in R (R Core Team, 2015). We used the ML-estimator. *T*-test were executed in conjunction with the Satterthwaite approximations to degrees of freedom.

## Results

### First research question: disentangling overall- and yearly effects of the three dyadic coping facets on relationship satisfaction

Tables 1 to 3 depict the estimates of the three random effects models disentangling timely stable from situational influences. For OSDC (Table 1), fixed effects reveal that the time specific (yearly) relationship satisfaction can be predicted by the general level of OSDC of both partners (level-2 actor effects of *γ* = 0.26 for female partners (ff; effect from female predictor to female outcome) and *γ* = 0.25 for male partners (mm; effect from male predictor to female outcome) as well as partner effects of *γ* = 0.22 (mf; effect from male predictor to female outcome) and *γ* = 0.15 (fm; effect from female predictor to male outcome). These effects are roughly double the size of yearly influences as depicted by level-1 effects (actor effects: *β* = 0.11 (ff) and *β* = 0.14 (mm) as well as partner effects: *β* = 0.12 (mf) and *β* = 0.06 (fm)). Since level-1 and level-2 variables are measured in identical scales, these effects may directly be compared indicating that level 2 (overall) associations are more pronounced than level 1 (yearly) associations.

**Table 1:** Random effects model predicting relationship satisfaction with OSDC.

	Female Partner				Male Partner		
	Estimate	*S*.*E*.	*p*		Estimate	*S*.*E*.	*P*
Level-1 (within-person) Main Effects (*β*)							
Intercept	**4.02**	0.03	< .01	Intercept	**4.04**	0.02	< .01
OSDC (a); ff	**0.11**	0.02	< .01	OSDC (a); mm	**0.14**	0.03	< .01
OSDC (p); mf	**0.12**	0.03	< .01	OSDC (p); fm	**0.06**	0.03	.03
Level-2 (between-person) Main Effects (*γ*)							
OSDC (a); ff	**0.26**	0.06	< .01	OSDC (a); mm	**0.25**	0.06	< .01
OSDC (p); mf	**0.22**	0.07	< .01	OSDC (p); fm	**0.15**	0.05	< .01

Estimate: estimated effect. *S*.*E*.: standard error. a: actor effect, p: partner effect.

OSDC: Own Supportive Dyadic Coping. Significant parameters are presented in bold type. ff: effect of female partners’ behavior on female partners’ outcome; fm: effect of female partners’ behavior on male partners’ outcome (mm and mf, accordingly)

**Table 2:** Random effects model predicting relationship satisfaction with PSDC.

	Female Partner				Male Partner		
	Estimate	*S*.*E*.	*p*		Estimate	*S*.*E*.	*p*
Level-1 (within-person) Main Effects (*β*)							
**Intercept**	**4.03**	**0.02**	**< .01**	**Intercept**	**4.04**	**0.02**	**< .01**
**PSDC (a); fm**	**0.21**	**0.03**	**< .01**	**PSDC (a); mf**	**0.17**	**0.02**	**< .01**
**PSDC (p); ff**	**0.07**	**0.03**	**< .01**	**PSDC (p); mm**	**0.08**	**0.02**	**< .01**
Level-2 (between-person) Main Effects (*γ*)							
**PSDC (a); fm**	**0.38**	**0.03**	**< .01**	**PSDC (a); fm**	**0.28**	**0.03**	**< .01**
**PSDC (p); ff**	**0.17**	**0.04**	**< .01**	**PSDC (p); mm**	**0.16**	**0.03**	**< .01**

Estimate: estimated effect. *S*.*E*.: standard error. a: actor effect, p: partner effect.

PSDC: Perceived Supportive Dyadic Coping provided by the partner.

Significant parameters are presented in bold type. ff: effect of female partners’ behavior on female partners’ outcome; fm: effect of female partners’ behavior on male partners’ outcome (mm and mf, accordingly)

**Table 3:** Random effects model predicting relationship satisfaction with CDC.

	Female Partner				Male Partner		
	Estimate	*S*.*E*.	*p*		Estimate	*S*.*E*.	*p*
Level-1 (within-person) Main Effects (*β*)							
Intercept	**4.03**	0.02	< .01	Intercept	**4.04**	0.02	< .01
CDC (a); ff	**0.15**	0.03	< .01	CDC (a); mm	**0.17**	0.03	< .01
CDC (p); mf	**0.10**	0.02	< .01	CDC (p); fm	**0.09**	0.02	< .01
Level-2 (between-person) Main Effects (*γ*)							
CDC (a); ff	**0.40**	0.04	< .01	CDC (a); mm	**0.29**	0.04	< .01
CDC (p); mf	**0.13**	0.05	.03	CDC (p); fm	**0.14**	0.04	< .01

Estimate: estimated effect. *S*.*E*.: standard error. a: actor effect, p: partner effect.

CDC: Common Dyadic Coping. Significant parameters are presented in bold type. ff: effect of female partners’ behavior on female partners’ outcome; fm: effect of female partners’ behavior on male partners’ outcome (mm and mf, accordingly)

For PSDC (Table 2), we find comparable results: level-2 effects are more pronounced than level-1 effects (actor effects: *γ* = 0.38 (mf) and *γ* = 0.28 (fm) as well as partner effects: *γ* = 0.17 (ff) and *γ* = 0.16 (mm)). Level-1 effects are approximately half the size as level-2 effects (actor effects: *β* = 0.21 (mf) and *β* = 0.17 (fm) as well as partner effects: *β* = 0.07 (ff) and *β* = 0.08 (mm)). Importantly, in these analyses, actor effects represent the effect the partner's behavior exerts on the actor's relationship satisfaction due to the fact that one partner's perceptions of the other partner's behavior serve as independent variables. That is, female (male) actor effects depict how the male (female) supportive dyadic coping (as rated by the female/male) partner) predicts female (male) partners' relationship satisfaction.

In terms of CDC (Table 3), level-2 effects are also more pronounced than level-1 effects (level-2 actor effects: *γ* = .40 (ff) and *γ* = 0.29 (mm); level-2 partner effects: *γ* = 0.13 (mf) and *γ* = 0.14 (fm)). At level-1, the results indicate significant actor and partner effects for both males and female, but these effects are lower compared to level-2 effects (level-1 actor effects: *β* = 0.15 (ff) and *β* = 0.17 (mm); level-1 partner effects: *β* = 0.10 (mf) and *β* = 0.09 (fm)).

To sum up, the results indicate significant level-1 (yearly) and level-2 (overall) associations of the two individual dyadic coping facets (OSDC and PSDC) and CDC with relationship satisfaction. This indicates that partners who report both higher overall and yearly dyadic coping (OSDC, PSDC, and CDC) are more satisfied with their relationships. The level 2 effects are approximately double in size than the level-1 effects, suggesting that overall levels of dyadic coping across years (OSDC, PSDC, and CDC) are better predictors of relationship satisfaction than yearly levels.

### Second research question: Differential effects of the three dyadic coping facets on relationship satisfaction

In order to test the differential effects of the three dyadic coping facets (see Table 4), we employed analyses with all three facets as predictors in one random effects model. In contrast to the models estimated for research question 1, we could only allow for random effects for level-1 actor effects due to the large number of random effects. Most interestingly, level-1 and level-2 fixed effects failed to reach significance for male and female OSDC at level-1. Specifically, the effects of yearly male and female OSDC on relationship satisfaction are not significant. In terms of the association between overall OSDC and relationship satisfaction (level-2), the results indicate that if women permanently provide support, their own (actor effect for female partners) and their partner’s relationship satisfaction (partner effect for male partners) are lower. For PSDC, we find significant level-1 effects except for the partner effect for women, and significant level-2 effects except for the actor effect for women. That is, yearly male and female PSDC predict his/her own relationship satisfaction (actor effects), but only male yearly PSDC predict female relationship satisfaction (partner effects). In addition, overall male PSDC predict his own relationship satisfaction (actor effect) and overall male and female PSDC predict his/her own relationship satisfaction (partner effects). In terms of CDC, the results show significant level 1- effects for both male and female (actor and partner effects), indicating that yearly CDC has a beneficial impact on relationship satisfaction. Level-2 effects are also significant for female partners (both actor and partner effects) and for male partners (only actor effects). This suggests that overall, timely stable CDC across years predicts relationship satisfaction, except partner effects for males.

**Table 4:** Random effects model predicting relationship satisfaction with OSDC, PSDC, and CDC.

	Female Partner				Male Partner		
	Estimate	*S*.*E*.	*p*		Estimate	*S*.*E*.	*p*
Level-1 (within-person) Main Effects (*β*)							
**Intercept**	**4.03**	**0.02**	**< .01**	**Intercept**	**4.04**	**0.02**	**< .01**
OSDC (a); ff	-0.02	0.02	.39	OSDC (a); mm	0.05	0.03	.09
OSDC (p); mf	0.03	0.03	.27	OSDC (p); fm	-0.02	0.02	.38
**PSDC (a)**; mf	**0.17**	**0.02**	**< .01**	**PSDC (a)**; fm	**0.12**	**0.02**	**< .01**
PSDC (p); ff	0.02	0.02	.35	**PSDC (p)**; mm	**0.05**	**0.02**	**.01**
**CDC (a)**; ff	**0.10**	**0.03**	**< .01**	**CDC (a)**; mm	**0.13**	**0.02**	**< .01**
**CDC (p)**; mf	**0.08**	**0.02**	**< .01**	**CDC (p)**; fm	**0.08**	**0.02**	**< .01**
Level-2 (between-person) Main Effects (*γ*)							
**OSDC (a)**; ff	**-0.17**	**0.06**	**< .01**	OSDC (a); mm	-0.10	0.06	.06
OSDC (p); mf	-0.09	0.06	.39	**OSDC (p)**; fm	**-0.12**	**0.05**	**.01**
PSDC (a); mf	0.34	0.04	.27	**PSDC (a)**; fm	**0.24**	**0.04**	**< .01**
**PSDC (p**); ff	**0.17**	**0.04**	**< .01**	**PSDC (p)**; mm	**0.18**	**0.04**	**< .01**
**CDC (a)**; ff	**0.20**	**0.05**	**< .01**	**CDC (a)**; mm	**0.19**	**0.05**	**< .01**
**CDC (p)**; mf	**0.08**	**0.06**	**< .01**	CDC (p); fm	0.04	0.05	.43

Estimate: estimated effect. *S*.*E*.: standard error. a: actor effect, p: partner effect.

OSDC: Own Supportive Dyadic Coping. PSDC: Perceived Supportive Dyadic Coping provided by the partner.

CDC: Common Dyadic Coping. Significant parameters are presented in bold type. ff: effect of female partners’ behavior on female partners’ outcome; fm: effect of female partners’ behavior on male partners’ outcome (mm and mf, accordingly)

In sum, the effects depict that the perceived support of one’s partner (PSDC) and shared support (CDC) seem to be more important for both partner’s relationship satisfaction compared to the effects of own provided support (OSDC) which is even detrimental for both partners if women show high scores of support provision across the years. These results underline the importance of perceiving support or sharing of coping behaviors rather than providing support. We reran the analyses employing only OSDC or PSDC in combination with CDC in order to prevent a statistical artifact due to collinearity. More exactly, we tested if coefficients representing effects of OSDC or PSDC did not reach significance because the same behavior is assessed twice but from the two partners’ perspectives (e.g., female OSDC assesses the same behavior as male PSDC) and hence non-significant estimates may be artificially produced. Yet, for the two analyses the same pattern was found.

### Third research question: The moderating role of stress

With respect to the third research question, we only report the final models. Tables 5 to 7 present the final estimates. Please remember that level-2 predictors are grand-mean centered and level-1 predictors are person-mean centered, hence, the interpretation of main effects hold under the condition that participants have mean scores on all other variables. For the first model (OSDC, Table 5), we find that for female partners yearly stress does not predict relationship satisfaction per se (level-1 effects), but for male partners it does. OSDC exerts a main effect for both partners as actor and partner effects at level-1. At level-2, the results indicate significant actor effects for female and significant actor and partner effects for male partners. Specifically, female overall stress predicts their own, but not their partner’s relationship satisfaction, while male overall stress predicts both their own and their partner’s relationship satisfaction. In addition, both partners’ overall OSDC promotes relationship satisfaction (actor and partner effects, level 2). Interestingly, the level-1 interactions of stress with OSDC are opposite in sign across genders. While more own stress and more OSDC at the moment leads to lower relationship satisfaction for women, it leads to higher relationship satisfaction for men. The results indicate that interactions, which turned significant at level-2 if entered separately, did not reach significance in the final model; the same is true for the cross-level interaction of momentary stress with general stress.

**Table 5:** Random effects model predicting relationship satisfaction with Stress and OSDC.

	Female Partner				Male Partner		
	Estimate	*S*.*E*.	*p*		Estimate	*S*.*E*.	*p*
Level-1 (within-person) Main Effects (*β*)							
Intercept	**4.02**	0.03	< .01	Intercept	**4.04**	0.02	< .01
Stress (a); ff	-0.08	0.04	.06	Stress (a); mm	**-0.09**	0.04	.04
Stress (p); mf	-0.02	0.04	.66	Stress (p)	**0.07**	0.04	.04
OSDC (a); ff	**0.09**	0.02	< .01	OSDC (a); mm	**0.14**	0.03	< .01
OSDC (p); mf	**0.12**	0.03	< .01	OSDC (p)	0.05	0.02	.03
Level-2 (between-person) Main Effects (*γ*)							
Stress (a); ff	**-0.44**	0.09	< .01	Stress (a); mm	**-0.22**	0.07	< .01
Stress (p); mf	0.04	0.08	.65	Stress (p)	**-0.15**	0.07	.04
OSDC (a); ff	**0.27**	0.06	< .01	OSDC (a); mm	**0.20**	0.06	< .01
OSDC (p); mf	**0.21**	0.07	< .01	OSDC (p)	**0.18**	0.05	< .01
Level-1 (within-person) Interactions							
Stress (a) x OSDC (a)	**-0.41**	0.12	< .01	Stress (a) x OSDC (a)	**0.32**	0.13	.02
Level-2 (between-person) Interactions							
Stress (a) x OSDC (a)	-0.08	0.15	.59	Stress (a) x OSDC (a)	-0.13	0.15	.42
Stress (p) x OSDC (a)	0.06	0.15	.72	Stress (p) x OSDC (a)	0.11	0.17	.53
Stress (a) x OSDC (p)	0.22	0.20	.28	Stress (a) x OSDC (p)	-0.09	0.13	.51
Stress (p) x OSDC (p)	-0.18	0.18	.33	Stress (p) x OSDC (p)	0.14	0.13	.25
Cross-Level-Interactions							
Stress L1 (a) x Stress L2 (p)	-0.08	0.13	0.53	Stress L1 (a) x Stress L2 (p)	-0.11	0.11	.34

Estimate: estimated effect. *S*.*E*.: standard error. a: actor effect, p: partner effect. L1: level-1; L2: level-2.

OSDC: Own Supportive Dyadic Coping. Significant parameters are presented in bold type. ff: effect of female partners’ behavior on female partners’ outcome; fm: effect of female partners’ behavior on male partners’ outcome (mm and mf, accordingly)

**Table 6:** Random effects model predicting relationship satisfaction with Stress and PSDC.

	Female Partner				Male Partner		
	Estimate	*S*.*E*.	*p*		Estimate	*S*.*E*.	*p*
Level-1 (within-person) Main Effects (*β*)							
Intercept	**4.03**	0.02	< .01	Intercept	**4.04**	0.02	< .01
Stress (a); ff	**-0.10**	0.04	.01	Stress (a); mm	**-0.11**	0.04	< .01
Stress (p); mf	-0.03	0.04	.48	Stress (p); fm	0.04	0.03	.25
PSDC (a); mf	**0.22**	0.03	< .01	PSDC (a); fm	**0.16**	0.02	< .01
PSDC (p); ff	**0.06**	0.02	< .01	PSDC (p); mm	**0.09**	0.02	< .01
Level-2 (between-person) Main Effects (*γ*)							
Stress (a); ff	**-0.31**	0.07	< .01	Stress (a); mm	-0.07	0.06	.25
Stress (p); mf	0.02	0.07	.79	Stress (p); fm	**-0.24**	**0.06**	**< .01**
PSDC (a); mf	**0.38**	0.03	< .01	PSDC (a); fm	**0.02**	**0.03**	**< .01**
PSDC (p); ff	**0.17**	0.04	< .01	PSDC (p); mm	**0.29**	**0.03**	**< .01**
Level-1 (within-person) Interactions							
Stress (a) x PSDC (a)	-0.11	0.10	.28	Stress (a) x PSDC (a)	**0.31**	0.10	< .01
Level-2 (between-person) Interactions							
Stress (a) x PSDC (a)	0.06	0.09	.48	Stress (a) x PSDC (a)	0.03	0.08	.70
Stress (p) x PSDC (a)	-0.05	0.10	.62	Stress (p) x PSDC (a)	0.16	0.08	.06
Stress (a) x PSDC (p)	0.10	0.09	.25	Stress (a) x PSDC (p)	-0.02	0.09	.80
Stress (p) x PSDC (p)	-0.04	0.09	.67	Stress (p) x PSDC (p)	-0.03	0.08	.73
Cross-Level-Interactions							
Stress L1 (a) x PSDC L2 (a)	0.01	0.05	.92	Stress L1 (a) x PSDC L2 (a)	**0.14**	0.06	.02
PSDC L1 (a) x PSDC L2(a)	**-0.13**	0.04	< .01	PSDC L1 (a) x PSDC L2(a)	**-0.09**	0.04	.01
PSDC L1 (a) x Stress L2 (a)	**0.19**	0.07	.01	PSDC L1 (a) x Stress L2 (a)	**0.15**	0.07	.04
PSDC L1 (a) x Stress L2 (p)	0.06	0.08	.46	PSDC L1 (a) x Stress L2 (p)	0.10	0.06	.12

Estimate: estimated effect. *S*.*E*.: standard error. a: actor effect, p: partner effect. L1: level-1; L2: level-2.

PSDC: Perceived Supportive Dyadic Coping provided by the partner. Significant parameters are presented in bold type. ff: effect of female partners’ behavior on female partners’ outcome; fm: effect of female partners’ behavior on male partners’ outcome (mm and mf, accordingly)

**Table 7:** Random effects model predicting relationship satisfaction with stress and CDC.

	Female Partner				Male Partner		
	Estimate	*S*.*E*.	*p*		Estimate	*S*.*E*.	*p*
Level-1 (within-person) Main Effects (*β*)							
Intercept	**4.03**	0.02	< .01	Intercept	**4.05**	0.02	< .01
Stress (a); ff	**-0.10**	0.04	< .01	Stress (a); mm	**-0.12**	0.04	< .01
Stress (p); mf	-0.03	0.04	.34	Stress (p); fm	0.05	0.03	.11
CDC (a); ff	**0.16**	0.03	< .01	CDC (a); mm	**0.17**	0.03	< .01
CDC (p); mf	**0.10**	0.02	< .01	CDC (p); fm	**0.11**	0.02	< .01
Level-2 Main Effects (*γ*)							
Stress (a); ff	**-0.33**	0.07	< .01	Stress (a); mm	**-0.22**	0.06	< .01
Stress (p); mf	0.04	0.07	.58	Stress (p); fm	-0.06	0.07	.36
CDC (a); ff	**0.41**	0.04	< .01	CDC (a); mm	**0.29**	0.04	< .01
CDC (p); mf	**0.11**	0.05	.03	CDC (p); fm	**0.13**	0.04	< .01
Level-2 (between-person) Interactions							
Stress (a) x CDC (a)	0.14	0.09	.15	Stress (a) x CDC (a)	< 0.01	0.10	1.00
Stress (p) x CDC (a)	-0.15	0.10	.13	**Stress (p) x CDC (a)**	**0.26**	0.10	.01
Cross-Level Interactions							
CDC L1 (a) x Stress L2 (a)	0.15	0.08	.07	CDC L1 (a) x Stress L2 (a)	-0.03	0.08	.69
CDC L1 (p) x Stress L2 (a)	**0.17**	0.07	.01	CDC L1 (p) x Stress L2 (a)	0.10	0.07	.15
CDC **L1** (a) x Stress L2 (p)	0.14	0.09	.12	CDC L1 (a) x Stress L2 (p)	**0.20**	0.07	.01
CDC **L1** (p) x Stress L2 (p)	-0.08	0.07	.22	CDC L1 (p) x Stress L2 (p)	**0.13**	0.06	.03

Estimate: estimated effect. *S*.*E*.: standard error. a: actor effect, p: partner effect. L1: level-1; L2: level-2.

CDC: Common Dyadic Coping. Significant parameters are presented in bold type.

ff: effect of female partners’ behavior on female partners’ outcome; fm: effect of female partners’ behavior on male partners’ outcome (mm and mf, accordingly)

With respect to CDC (Table 7), we find the same pattern of actor and partner effects that already emerged for stress and CDC at level-1 and level-2 reported in Table 3. Regarding the interaction terms, we find a significant interaction of the overall stress level of female partners and CDC at level-2 for male partners in such a way that more stress of female partners and more CDC reported by men lead to principally higher relationship satisfaction in male partners (*γ* = 0.26). Additionally, CDC reported by men in a specific year leads to higher relationship satisfaction in women when female partners report higher levels of overall stress (*γ* = 0.17); for male partners, we find that they are more satisfied when they report more CDC in a specific year and their partners are generally more stressed. Thus, the male and female partner of a couple with a permanently stressed female partner seem to be more satisfied with their relationship in moments when they have higher levels of CDC.

For PSDC (Table 6), we find a comparable pattern of level-1 and level-2 main effects. Yet, the level-1 interaction of stress with PSDC did not reach significance in female partners but only in male partners. We did find cross-level interactions with negative effects of stable and momentary PSDC, reflecting the fact that those who generally perceive support by their partner and additionally perceive more support at the moment than usually do feel less satisfied (both partners). Being stressed at the moment but having a generally supportive partner is beneficial for male partners’ relationship satisfaction. Receiving more support at the moment than generally (PSDC L1) and being generally stressed also leads to higher relationship satisfaction.

In sum, we find more significant interaction effects of stress and dyadic coping facets considering those facets depicting perceived support (PSDC and CDC) in contrast to facets depicting providing support. Hence, activation of support in times of stress (at both between and within level) buffers the negative impact of stress on relationship satisfaction for the stressed partner.

## Discussion

The importance of dyadic coping on relationship satisfaction for couples confronting with stress has been revealed in previous studies; however specific hypotheses comparing different forms of dyadic coping and disentangling between- and within- level effects have been overlooked in past research. Guided by the Systemic Transactional Model (STM; [2]), the present study focused on examining the influence of provided and received supportive dyadic coping and common dyadic coping on relationship satisfaction in a longitudinal study and by analyzing stress as a moderator in these associations.

### Dyadic coping and relationship satisfaction: Between- and within-person variations

Our first research question aimed to disentangle between-person (overall, timely stable) from within-person (yearly, time specific) associations of own supportive dyadic coping, perceived supportive dyadic coping provided by the partner, and common dyadic coping with relationship satisfaction. Results revealed significant between- and within-person effects of all three forms of dyadic coping on relationship satisfaction. Thus, men and women were more satisfied with their relationship when they themselves and their partners reported higher levels of dyadic coping (ODC, PSDC, CDC) both overall (timely stable) and yearly (time specific).

Regarding the association of own supportive dyadic coping with relationship satisfaction, our results are supported by studies indicating that providing support and care to others is an inherent need and has a positive impact on psychological and physical well-being of both provider and recipient [4,23–25]. Furthermore, our findings are consistent with the Conceptual Model for Thriving through Relationships [7], which emphasizes that both giving and receiving support predicts positive relationship outcomes, such as relationship satisfaction, which, in turn, will affect the provider’s thriving and well-being. People benefit not only from receiving, but also from giving to others (e.g., giving support, time, money). Specifically, autonomy support given to a friend in a close friendship has been positively related to adjustment in friendship and well-being for both provider an recipient [24], providing support to a partner has been related to provider’s feelings of social connection and reduced physiological stress response [49], pro-social spending of money has been found to be more related to provider’s happiness than personal spending [50], and providing emotional support to spouses has been positively related to longevity in older adults and better physical health [23,51]. The mechanisms explaining the aforementioned associations may be related to feelings of usefulness to others and helpfulness, purpose and meaning, positive self-esteem and social well-being of the providers when they are able to give support to a loved one.

The findings indicating significant between-person positive effects of supportive dyadic coping provided by the partner on relationship satisfaction are in line with the results reported in prior studies [4,10,21]. In addition, the within-person, year-specific effects found in the present longitudinal study regarding the association between perceived supportive dyadic coping provided by the partner and relationship satisfaction are consistent with results reported in previous daily diary studies, which showed positive associations of daily received support with relationship satisfaction, closeness, and partners’ positive emotions [17–19]. That is, fluctuations of dyadic coping do not only affect day-specific relationship satisfaction but also year-specific judgments of relationship satisfaction. Our results complement existing research by investigating both own supportive dyadic coping and perceived supportive dyadic coping provided by the partner and by analyzing both between- and within-person associations of dyadic coping with relationship satisfaction longitudinally.

The findings showing positive associations of common dyadic coping with relationship satisfaction at both between- and within-persons level are consistent with previous studies conducted with clinical samples, which indicated that common dyadic coping has been related to positive outcomes in couples with one partner suffering from diabetes, cancer, depression, or anxiety [6,12,22,31]. Therefore, common dyadic coping is important for relationship satisfaction of couples from both community and clinical samples.

Interestingly, for all forms of dyadic coping, the predictive effects of overall support were approximately double in size compared to the predictive effects of yearly support. Considering that the current study included couples in long-term relationships, a possible explanation of this finding might reside in the fact that for these partners overall level of support and consistency in support may be more important than time specific changes in their own and their partners’ supportive behavior. Another possible explanation of these results may be related with the difference between *perceived available support* and *received support* [52]. Some studies showed that perceived available support is a better predictor of mental health outcomes than received support [7]. Therefore, in the context of the present study, overall perceived available dyadic coping (average level of dyadic coping) is more important for relationship satisfaction than time specific dyadic coping (dyadic coping in specific years).

### Comparing the effects of the three facets of dyadic coping on relationship satisfaction

In our second research question we compared the overall and yearly effects of own supportive dyadic coping, perceived supportive dyadic coping provided by the partner, and common dyadic coping on relationship satisfaction and aimed to test, which form of dyadic coping is more important compared to the others. Analyses indicated that overall and yearly own supportive dyadic coping is less important for both partner’s relationship satisfaction compared to the overall and yearly effects of perceived supportive dyadic coping provided by the partner and common dyadic coping. These results indicating a stronger effect of perceived supportive dyadic coping provided by the partner fit with the studies showing that receiving support is a better predictor of relationship satisfaction than providing support [4,53]. One explanation for this finding may be that providing support for longer periods may deplete personal resources of the provider and may be stressful [27,53]. Although giving to others and providing support was associated with positive outcomes, receiving support (both within and between level) seems to be more important for relationship satisfaction of both partners. Future research should explore the effects of providing support on both relational and individual outcomes, as it may have negative consequences on individual outcomes (e.g., emotional resources), but positive effects on relationship outcomes (e.g., relationship satisfaction).

That common dyadic coping did not reveal to be a stronger predictor of relationship satisfaction than perceived supportive dyadic coping provided by the partner, as repeatedly found in studies on couples dealing with severe illness may have two reasons. First, in everyday life stressors often concern one partner first and only subsequently become a dyadic stressor, thus partners are more often engaging in supportive dyadic coping. According to the STM, common dyadic coping should be elicited mainly in situations where both partners are stressed at the same time by demands appraised as a shared issue requiring both partners’ coping efforts. In this study the type of stress (*indirect dyadic stress*: first individual and then becoming dyadic, going along with supportive dyadic coping vs. *direct dyadic stress*: affecting both partners at the same time eliciting common dyadic coping) was not assessed, therefore limiting the explanative power of this comparison. Future research might benefit further from differentiating the source of stress and analyzing what type of coping is more beneficial in the case of indirect dyadic stress in comparison with direct dyadic stress.

### The role of stress

In our third research question we examined the role of yearly and overall stress for understanding the association between dyadic coping and relationship satisfaction. Overall, the association between own supportive dyadic coping and relationship satisfaction was moderated to a lesser degree by stress compared to the associations of perceived supportive dyadic coping provided by the partner and common dyadic coping with relationship satisfaction. As expected, most interaction effects indicated that the effect of dyadic coping on relationship satisfaction is intensified when one partner is stressed. These results indicate that mainly perceived supportive dyadic coping provided by the partner and common dyadic coping play a crucial role for relationship satisfaction of stressed couples and are more important than own supportive dyadic coping. Despite its positive influence on both the provider and recipient, support-provision might be demanding especially in stressful moments because it requires emotional and cognitive resources, specific skills (e.g., empathy, perspective-taking, emotions regulation skills), and altruistic motivation [7].

In terms of own supportive dyadic coping, the only significant interaction effect with stress revealed that in moments with higher own stress and higher own supportive dyadic coping, women are less satisfied, and men are more satisfied with their relationship. The gender difference might be explained by gender role. As women provide more support in general and feel more responsible and obligated to do this [54], they might experience a depletion of personal resources and burnout when providing even more support to their partners in highly stressful situations, so that their momentary relationship satisfaction might decrease.

For perceived supportive dyadic coping provided by the partner, results indicated that men with more time-specific stress were more satisfied with their relationship when they perceived more time-specific and more overall support by their female partners. This finding aligns with previous studies showing that women are more responsive to their partners’ stress and are more efficient than men in providing support on days when their partners experience higher levels of stress [54]. Additionally, our study revealed that men and women who were permanently stressed were more satisfied with their relationship in moments when they perceived more support by their partners than usual. These results replicate the findings of a recent daily diary study, showing that both partners reported higher levels of relationship satisfaction on days when they received more supportive dyadic coping by partner [18].

Interestingly, men and women generally perceiving strong support by their partner were less satisfied with their relationship in moments when they perceived additional support to their usual support received. This may indicate that an overprovision of partner support could have negative consequences [37]. This finding converges with prior studies showing that receiving unwanted support and depending on the partner for support have been related to feelings of inefficacy, incompetence, negative emotions, and low self-esteem of the receivers [17,26,27]. Moreover, other studies emphasized the importance of measuring support adequacy [55], support equity and reciprocity (i.e., equity between support provided and received) [17], and autonomy support (i.e., providing support but accepting partner’s perspective and encouraging their choices, rather than support frequency) [25]. In addition, this finding provides support to the Social Support Effectiveness Model [30], which posits that support provided should match the needs of the recipients in terms of quantity and quality. Future studies are needed to disentangle the interplay between support frequency, support adequacy, and autonomy support in predicting relationship satisfaction longitudinally.

With regard to common dyadic coping, results suggested that couples with permanently stressed women were more satisfied with their relationship when partners reported higher common dyadic coping. The effect was particularly consistent for men’s relationship satisfaction. This result is consistent with prior studies showing that common dyadic coping is particularly important for women coping with stress, influencing both their own and their partners relationship satisfaction [36] and relationship quality [13].

### Strengths and limitations

The present study has a number of conceptual and methodological strengths: (a) comparing the effects of different forms of dyadic coping on relationship satisfaction by using data collected from a community sample; (b) disentangling timely stable from time-specific fluctuations in the associations of dyadic coping with relationship satisfaction and (c) using a longitudinal design with five measurement points, which enabled us to analyze changes in couple relationships. However, there are a few limitations that should be mentioned. The present study included well-educated, middle class-couples from a developed Western country and dropouts specific to longitudinal studies, which limits the generalizability of our findings. In particular, we only analyzed data from couples being willing to participate in a longitudinal study and having a stable relationship across the five occasions of measurement. We did not analyze the longitudinal data of couples who separated nor of those who dropped out for unknown reasons. Future research is needed on couples from different cultures and therefore experiencing different levels of stress in order to better understand the interplay between dyadic coping and relationship satisfaction. Moreover, generalizability of our findings is limited to heterosexual couples. Future studies should analyze the association between stress, dyadic coping and relationship satisfaction in same-sex couples, who might experience additional stressors, such as sexual minority stress. Another limitation related to data collection is that participants completed questionnaires only once a year and these yearly momentary measures have been compared to the global, measures of the study variables. Daily diary designs are necessary in order to analyze data collected in more measurement points for a better disaggregation of the effects on the between- and within-person level. Furthermore, future studies should focus on replacing laboratory settings with measurements in real life conditions. For example, Ecological Momentary Assessment (EMA, daily intensive repeated measurements) is a promising methodology for understanding interpersonal processes in couple research [56] and may be used to examine within-person relationship and temporal dynamics in future studies investigating coping processes in association with relationship outcomes and stress.

## Conclusions and implications

The results of the current research study suggest the importance of addressing both between- and within- person fluctuations in supportive dyadic coping (support provided to and received from the partner) and common dyadic coping in future research studies, as well as in couple therapy and simultaneously considering the level of stress experienced by partners. First, the findings of the present study revealed that between-person effects of all three forms of dyadic coping have been more strongly related to relationship satisfaction than within-person effects. This indicates that a generally high level of dyadic coping across several years is more important for relationship satisfaction compared to momentary fluctuations from year to year. Constant support and perception of support availability seem to be more important for couples in long-term relationships than momentary changes in dyadic coping.

Second, our results suggest that receiving supportive dyadic coping and common dyadic coping are better predictors of relationship satisfaction than providing support. Despite its positive consequences, providing support may have negative consequences especially for women’s relationships satisfaction, possibly through depleting their resources. Support reciprocity may be more effective than only giving or receiving support. In addition, the results of our study emphasize the importance of common dyadic coping for relationship satisfaction of both partners and especially for women in highly stressful moments. The benefits of dyadic coping may also depend on the cultural context and future studies should compare provided, received, and common dyadic coping in couples belonging to different cultures.

Third, couples confronting to stressful situations may benefit more in their relationship from supportive dyadic coping provided by the partner and common dyadic coping than from own supportive dyadic coping, as providing support may be too demanding. In addition, despite the importance of supportive dyadic coping received by partners for relationship satisfaction, additional support for couples that already experience a high level of overall dyadic coping may have negative consequences.

Finally, therapists working with couples and using validated relationship education programs (e.g., Couples Coping Enhancement Training, CCET) [57] are encouraged to address issues related to support reciprocity and support efficiency in couples coping with different levels of stress. It is necessary to emphasize that constant supportive and common dyadic coping provided to the partner are more important for long term relationships than momentary dyadic coping, giving support and not only receiving it has positive consequences for both provider and recipient, and an overprovision of support may have negative consequences by determining feelings of inefficacy; thus, support equity should be promoted. Moreover, based on our findings, researchers should focus on measuring perceived supportive dyadic coping provided by the partner and common dyadic coping, rather than own dyadic coping.

## Supporting information

S1 Fig Flow chart of the stepwise analysis procedure for research question 3 with Own Supportive Dyadic Coping (OSDC) as exemplary independent variable. Step 1: determining the necessary random effects structure with stress as predictor at level-1 and level-2. Step 2: Additionally, integrating cross-level interactions of stress. Step 3: Entering main effects of OSDC at level-1 and level-2. Step 4: incorporating interactions of stress with OSDC separately for level-1 interactions, level-2 interactions, cross-level interactions due to the complexity of the models. Step 5: model with stress at level-1 and level-2, cross-level interaction of stress, main effects of OSDC, and all interaction effects of step 4 that turned significant when entered separately.
(TIF)

S1 Table Random effects model predicting relationship satisfaction with OSDC. (PDF)

S2 Table Random effects model predicting relationship satisfaction with PSDC. (PDF)

S3 Table Random effects model predicting relationship satisfaction with CDC. (PDF)

S4 Table Random effects model predicting relationship satisfaction with OSDC, PSDC, and CDC. (PDF)

S5 Table Random effects model predicting relationship satisfaction with stress and OSDC. (PDF)

S6 Table Random effects model predicting relationship satisfaction with stress and PSDC. (PDF)

S7 Table Random effects model predicting relationship satisfaction with stress and CDC. (PDF)

S1 Appendix List of previous publications based on the data used in the present article. (DOCX)
