# Supplementary figures and images for: Stress, dyadic coping, and relationship satisfaction: A longitudinal study disentangling timely stable from yearly fluctuations
Source: PLoS One. 2020 Apr 9;15(4):e0231133. doi: 10.1371/journal.pone.0231133 (PMC7145192; doi:10.1371/journal.pone.0231133)

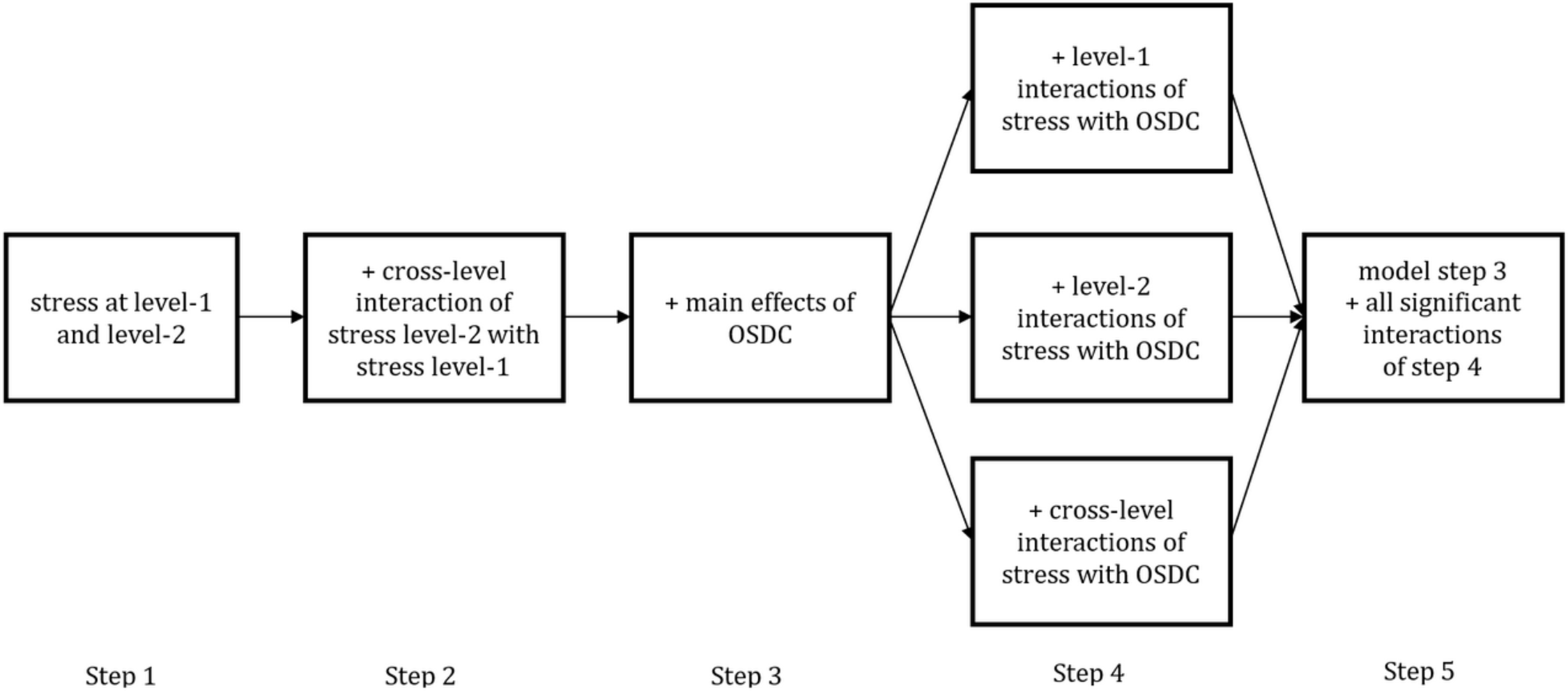

Supplement: S1 Fig — Step 1: determining the necessary random effects structure with stress as predictor at level-1 and level-2. Step 2: Additionally, integrating cross-level interactions of stress. Step 3: Entering main effects of OSDC at level-1 and level-2. Step 4: incorporating interactions of stress with OSDC separately for level-1 interactions, level-2 interactions, cross-level interactions due to the complexity of the models. Step 5: model with stress at level-1 and level-2, cross-level interaction of stress, main effects of OSDC, and all interaction effects of step 4 that turned significant when entered separately. (TIF) [file pone.0231133.s001.tif]
