# Supplementary material for: Stress, dyadic coping, and relationship satisfaction: A longitudinal study disentangling timely stable from yearly fluctuations
Source: PLoS One. 2020 Apr 9;15(4):e0231133. doi: 10.1371/journal.pone.0231133 (PMC7145192; doi:10.1371/journal.pone.0231133)
Supplement: S1 Table — (PDF) [file pone.0231133.s002.pdf]

S1Table. Random Effects Model Predicting Relationship Satisfaction with OSDC

	Female Partner					Male Partner		
	Estimate	*S.E.*	*p*			Estimate	*S.E.*	*p*
Level-1 (within-person) Main Effects (β)								
**Intercept**	**4.02**	**0.03**	**< .01**		**Intercept**	**4.04**	**0.02**	**< .01**
**OSDC (a)**	**0.11**	**0.02**	**< .01**		**OSDC (a)**	**0.14**	**0.03**	**< .01**
**OSDC (p)**	**0.12**	**0.03**	**< .01**		**OSDC (p)**	**0.06**	**0.03**	**.03**
Level-2 (between-person) Main Effects (γ)								
**OSDC (a)**	**0.26**	**0.06**	**< .01**		**OSDC (a)**	**0.25**	**0.06**	**< .01**
**OSDC (p)**	**0.22**	**0.07**	**< .01**		**OSDC (p)**	**0.15**	**0.05**	**< .01**

Notes. Estimate: estimated effect. *S.E.*: standard error. a: actor effect, p: partner effect.
OSDC: Own Supportive Dyadic Coping. Significant parameters are presented in bold type.
