# Supplementary material for: Stress, dyadic coping, and relationship satisfaction: A longitudinal study disentangling timely stable from yearly fluctuations
Source: PLoS One. 2020 Apr 9;15(4):e0231133. doi: 10.1371/journal.pone.0231133 (PMC7145192; doi:10.1371/journal.pone.0231133)
Supplement: S2 Table — (PDF) [file pone.0231133.s003.pdf]

S2 Table. Random Effects Model Predicting Relationship Satisfaction with PSDC

	Female Partner					Male Partner		
	Estimate	*S.E.*	*p*			Estimate	*S.E.*	*p*
Level-1 (within-person) Main Effects (β)								
**Intercept**	**4.03**	**0.02**	**< .01**		**Intercept**	**4.04**	**0.02**	**< .01**
**PSDC (a)**	**0.21**	**0.03**	**< .01**		**PSDC (a)**	**0.17**	**0.02**	**< .01**
**PSDC (p)**	**0.07**	**0.03**	**< .01**		**PSDC (p)**	**0.08**	**0.02**	**< .01**
Level-2 (between-person) Main Effects (γ)								
**PSDC (a)**	**0.38**	**0.03**	**< .01**		**PSDC (a)**	**0.28**	**0.03**	**< .01**
**PSDC (p)**	**0.17**	**0.04**	**< .01**		**PSDC (p)**	**0.16**	**0.03**	**< .01**

Notes. Estimate: estimated effect. *S.E.*: standard error. a: actor effect, p: partner effect.
PSDC: Perceived Supportive Dyadic Coping provided by the partner.
Significant parameters are presented in bold type.
