# Supplementary material for: Stress, dyadic coping, and relationship satisfaction: A longitudinal study disentangling timely stable from yearly fluctuations
Source: PLoS One. 2020 Apr 9;15(4):e0231133. doi: 10.1371/journal.pone.0231133 (PMC7145192; doi:10.1371/journal.pone.0231133)
Supplement: S3 Table — (PDF) [file pone.0231133.s004.pdf]

S3 Table. Random Effects Model Predicting Relationship Satisfaction with CDC

	Female Partner					Male Partner		
	Estimate	*S.E.*	*p*			Estimate	*S.E.*	*p*
Level-1 (within-person) Main Effects (β)								
**Intercept**	**4.03**	**0.02**	**< .01**		**Intercept**	**4.04**	**0.02**	**< .01**
**CDC (a)**	**0.15**	**0.03**	**< .01**		**CDC (a)**	**0.17**	**0.03**	**< .01**
**CDC (p)**	**0.10**	**0.02**	**< .01**		**CDC (p)**	**0.09**	**0.02**	**< .01**
Level-2 (between-person) Main Effects (γ)								
**CDC (a)**	**0.40**	**0.04**	**< .01**		**CDC (a)**	**0.29**	**0.04**	**< .01**
**CDC (p)**	**0.13**	**0.05**	**.03**		**CDC (p)**	**0.14**	**0.04**	**< .01**

Notes. Estimate: estimated effect. *S.E.*: standard error. a: actor effect, p: partner effect.
CDC: Common Dyadic Coping. Significant parameters are presented in bold type.
