# Supplementary material for: Stress, dyadic coping, and relationship satisfaction: A longitudinal study disentangling timely stable from yearly fluctuations
Source: PLoS One. 2020 Apr 9;15(4):e0231133. doi: 10.1371/journal.pone.0231133 (PMC7145192; doi:10.1371/journal.pone.0231133)
Supplement: S4 Table — (PDF) [file pone.0231133.s005.pdf]

S4 Table. Random Effects Model Predicting Relationship Satisfaction with OSDC, PSDC, and CDC

	Female Partner					Male Partner		
	Estimate	*S.E.*	*p*			Estimate	*S.E.*	*p*
Level-1 (within-person) Main Effects (β)								
**Intercept**	**4.03**	**0.02**	**< .01**		**Intercept**	**4.04**	**0.02**	**< .01**
OSDC (a)	-0.02	0.02	.39		OSDC (a)	0.05	0.03	.09
OSDC (p)	0.03	0.03	.27		OSDC (p)	-0.02	0.02	.38
**PSDC (a)**	**0.17**	**0.02**	**< .01**		**PSDC (a)**	**0.12**	**0.02**	**< .01**
PSDC (p)	0.02	0.02	.35		**PSDC (p)**	**0.05**	**0.02**	**.01**
**CDC (a)**	**0.10**	**0.03**	**< .01**		**CDC (a)**	**0.13**	**0.02**	**< .01**
**CDC (p)**	**0.08**	**0.02**	**< .01**		**CDC (p)**	**0.08**	**0.02**	**< .01**
Level-2 (between-person) Main Effects (γ)								
**OSDC (a)**	**-0.17**	**0.06**	**< .01**		OSDC (a)	-0.10	0.06	.06
OSDC (p)	-0.09	0.06	.39		**OSDC (p)**	**-0.12**	**0.05**	**.01**
PSDC (a)	0.34	0.04	.27		**PSDC (a)**	**0.24**	**0.04**	**< .01**
**PSDC (p)**	**0.17**	**0.04**	**< .01**		**PSDC (p)**	**0.18**	**0.04**	**< .01**
**CDC (a)**	**0.20**	**0.05**	**< .01**		**CDC (a)**	**0.19**	**0.05**	**< .01**
**CDC (p)**	**0.08**	**0.06**	**< .01**		CDC (p)	0.04	0.05	.43

Notes. Estimate: estimated effect. *S.E.*: standard error. a: actor effect, p: partner effect.
OSDC: Own Supportive Dyadic Coping. PSDC: Perceived Supportive Dyadic Coping provided by the partner.
CDC: Common Dyadic Coping. Significant parameters are presented in bold type.
