# Supplementary material for: Stress, dyadic coping, and relationship satisfaction: A longitudinal study disentangling timely stable from yearly fluctuations
Source: PLoS One. 2020 Apr 9;15(4):e0231133. doi: 10.1371/journal.pone.0231133 (PMC7145192; doi:10.1371/journal.pone.0231133)
Supplement: S5 Table — (PDF) [file pone.0231133.s006.pdf]

S5 Table. Random Effects Model Predicting Relationship Satisfaction with Stress and OSDC

	Female Partner					Male Partner		
	Estimate	*S.E.*	*p*			Estimate	*S.E.*	*p*
Level-1 (within-person) Main Effects (β)								
**Intercept**	**4.02**	**0.03**	**< .01**		**Intercept**	**4.04**	**0.02**	**< .01**
Stress (a)	-0.08	0.04	.06		**Stress (a)**	**-0.09**	**0.04**	**.04**
Stress (p)	-0.02	0.04	.66		**Stress (p)**	**0.07**	**0.04**	**.04**
**OSDC (a)**	**0.09**	**0.02**	**< .01**		**OSDC (a)**	**0.14**	**0.03**	**< .01**
**OSDC (p)**	**0.12**	**0.03**	**< .01**		**OSDC (p)**	**0.05**	**0.02**	**.03**
Level-2 (between-person) Main Effects (γ)								
**Stress (a)**	**-0.44**	**0.09**	**< .01**		**Stress (a)**	**-0.22**	**0.07**	**< .01**
Stress (p)	0.04	0.08	.65		**Stress (p)**	**-0.15**	**0.07**	**.04**
**OSDC (a)**	**0.27**	**0.06**	**< .01**		**OSDC (a)**	**0.20**	**0.06**	**< .01**
**OSDC (p)**	**0.21**	**0.07**	**< .01**		**OSDC (p)**	**0.18**	**0.05**	**< .01**
Level-1 (within-person) Interactions								
**Stress (a) x OSDC (a)**	**-0.41**	**0.12**	**< .01**		**Stress (a) x OSDC (a)**	**0.32**	**0.13**	**.02**
Level-2 (between-person) Interactions								
Stress (a) x OSDC (a)	-0.08	0.15	.59		Stress (a) x OSDC (a)	-0.13	0.15	.42
Stress (p) x OSDC (a)	0.06	0.15	.72		Stress (p) x OSDC (a)	0.11	0.17	.53
Stress (a) x OSDC (p)	0.22	0.20	.28		Stress (a) x OSDC (p)	-0.09	0.13	.51
Stress (p) x OSDC (p)	-0.18	0.18	.33		Stress (p) x OSDC (p)	0.14	0.13	.25
Cross-Level-Interactions								
Stress L1 (a) x Stress L2 (p)	-0.08	0.13	0.53		Stress L1 (a) x Stress L2 (p)	-0.11	0.11	.34

Notes. Estimate: estimated effect. *S.E.*: standard error. a: actor effect, p: partner effect. L1: level-1; L2: level-2.
OSDC: Own Supportive Dyadic Coping. Significant parameters are presented in bold type.
