# Supplementary material for: Stress, dyadic coping, and relationship satisfaction: A longitudinal study disentangling timely stable from yearly fluctuations
Source: PLoS One. 2020 Apr 9;15(4):e0231133. doi: 10.1371/journal.pone.0231133 (PMC7145192; doi:10.1371/journal.pone.0231133)
Supplement: S6 Table — (PDF) [file pone.0231133.s007.pdf]

S6 Table. Random Effects Model Predicting Relationship Satisfaction with Stress and PSDC

	Female Partner					Male Partner		
	Estimate	*S.E.*	*p*			Estimate	*S.E.*	*p*
Level-1 (within-person) Main Effects (β)								
**Intercept**	**4.03**	**0.02**	**< .01**		**Intercept**	**4.04**	**0.02**	**< .01**
**Stress (a)**	**-0.10**	**0.04**	**.01**		**Stress (a)**	**-0.11**	**0.04**	**< .01**
Stress (p)	-0.03	0.04	.48		Stress (p)	0.04	0.03	.25
**PSDC (a)**	**0.22**	**0.03**	**< .01**		**PSDC (a)**	**0.16**	**0.02**	**< .01**
**PSDC (p)**	**0.06**	**0.02**	**< .01**		**PSDC (p)**	**0.09**	**0.02**	**< .01**
Level-2 (between-person) Main Effects (γ)								
**Stress (a)**	**-0.31**	**0.07**	**< .01**		Stress (a)	-0.07	0.06	.25
Stress (p)	0.02	0.07	.79		**Stress (p)**	**-0.24**	**0.06**	**< .01**
**PSDC (a)**	**0.38**	**0.03**	**< .01**		**PSDC (a)**	**0.02**	**0.03**	**< .01**
**PSDC (p)**	**0.17**	**0.04**	**< .01**		**PSDC (p)**	**0.29**	**0.03**	**< .01**
Level-1 (within-person) Interactions								
Stress (a) x PSDC (a)	-0.11	0.10	.28		**Stress (a) x PSDC (a)**	**0.31**	**0.10**	**< .01**
Level-2 (between-person) Interactions								
Stress (a) x PSDC (a)	0.06	0.09	.48		Stress (a) x PSDC (a)	0.03	0.08	.70
Stress (p) x PSDC (a)	-0.05	0.10	.62		Stress (p) x PSDC (a)	0.16	0.08	.06
Stress (a) x PSDC (p)	0.10	0.09	.25		Stress (a) x PSDC (p)	-0.02	0.09	.80
Stress (p) x PSDC (p)	-0.04	0.09	.67		Stress (p) x PSDC (p)	-0.03	0.08	.73
Cross-Level-Interactions								
Stress L1 (a) x PSDC L2 (a)	0.01	0.05	.92		**Stress L1 (a) x PSDC L2 (a)**	**0.14**	**0.06**	**.02**
**PSDC L1 (a) x PSDC L2(a)**	**-0.13**	**0.04**	**< .01**		**PSDC L1 (a) x PSDC L2(a)**	**-0.09**	**0.04**	**.01**
**PSDC L1 (a) x Stress L2 (a)**	**0.19**	**0.07**	**.01**		**PSDC L1 (a) x Stress L2 (a)**	**0.15**	**0.07**	**.04**
PSDC L1 (a) x Stress L2 (p)	0.06	0.08	.46		PSDC L1 (a) x Stress L2 (p)	0.10	0.06	.12

Notes. Estimate: estimated effect. *S.E.*: standard error. a: actor effect, p: partner effect. L1: level-1; L2: level-2.
PSDC: Perceived Supportive Dyadic Coping provided by the partner. Significant parameters are presented in bold type.
