# Supplementary material for: Stress, dyadic coping, and relationship satisfaction: A longitudinal study disentangling timely stable from yearly fluctuations
Source: PLoS One. 2020 Apr 9;15(4):e0231133. doi: 10.1371/journal.pone.0231133 (PMC7145192; doi:10.1371/journal.pone.0231133)
Supplement: S7 Table — (PDF) [file pone.0231133.s008.pdf]

S7 Table. Random Effects Model Predicting Relationship Satisfaction with Stress and CDC

	Female Partner					Male Partner		
	Estimate	*S.E.*	*p*			Estimate	*S.E.*	*p*
Level-1 (within-person) Main Effects (β)								
**Intercept**	**4.03**	**0.02**	**< .01**		**Intercept**	**4.05**	**0.02**	**< .01**
**Stress (a)**	**-0.10**	**0.04**	**< .01**		**Stress (a)**	**-0.12**	**0.04**	**< .01**
Stress (p)	-0.03	0.04	.34		Stress (p)	0.05	0.03	.11
**CDC (a)**	**0.16**	**0.03**	**< .01**		**CDC (a)**	**0.17**	**0.03**	**< .01**
**CDC (p)**	**0.10**	**0.02**	**< .01**		**CDC (p)**	**0.11**	**0.02**	**< .01**
Level-2 Main Effects (γ)								
**Stress (a)**	**-0.33**	**0.07**	**< .01**		**Stress (a)**	**-0.22**	**0.06**	**< .01**
Stress (p)	0.04	0.07	.58		Stress (p)	-0.06	0.07	.36
**CDC (a)**	**0.41**	**0.04**	**< .01**		**CDC (a)**	**0.29**	**0.04**	**< .01**
**CDC (p)**	**0.11**	**0.05**	**.03**		**CDC (p)**	**0.13**	**0.04**	**< .01**
Level-2 (between-person) Interactions								
Stress (a) x CDC (a)	0.14	0.09	.15		Stress (a) x CDC (a)	< 0.01	0.10	1.00
Stress (p) x CDC (a)	-0.15	0.10	.13		**Stress (p) x CDC (a)**	**0.26**	**0.10**	**.01**
Cross-Level Interactions								
CDC L1 (a) x Stress L2 (a)	0.15	0.08	.07		CDC L1 (a) x Stress L2 (a)	-0.03	0.08	.69
**CDC L1 (p) x Stress L2 (a)**	**0.17**	**0.07**	**.01**		CDC L1 (p) x Stress L2 (a)	0.10	0.07	.15
CDC **L1** (a) x Stress L2 (p)	0.14	0.09	.12		**CDC L1 (a) x Stress L2 (p)**	**0.20**	**0.07**	**.01**
CDC **L1** (p) x Stress L2 (p)	-0.08	0.07	.22		**CDC L1 (p) x Stress L2 (p)**	**0.13**	**0.06**	**.03**

Notes. Estimate: estimated effect. *S.E.*: standard error. a: actor effect, p: partner effect. L1: level-1; L2: level-2.
CDC: Common Dyadic Coping. Significant parameters are presented in bold type.
