# Supplementary material for: Stress, dyadic coping, and relationship satisfaction: A longitudinal study disentangling timely stable from yearly fluctuations
Source: PLoS One. 2020 Apr 9;15(4):e0231133. doi: 10.1371/journal.pone.0231133 (PMC7145192; doi:10.1371/journal.pone.0231133)
Supplement: S1 Appendix — (DOCX) [file pone.0231133.s009.docx]

S8. Appendix. List of previous publications based on the data used in the present article

1. Backes S, Brandstätter V, Kuster M, Nussbeck FW, Bradbury TN, Bodenmann G, et al. Who suffers from stress? Action-state orientation moderates the effect of external stress on relationship satisfaction. Journal of Social and Personal Relationships. 2017 Sep;34(6):894–914.

2. Bernecker K, Ghassemi M, Brandstätter V. Approach and avoidance relationship goals and couples’ nonverbal communication during conflict. European Journal of Social Psychology [Internet]. 2018 May 15 [cited 2019 Mar 18]; Available from: http://doi.wiley.com/10.1002/ejsp.2379

3. Denzinger F, Backes S, Brandstätter V. Same same but different: Similarity of goals and implicit motives in intimate relationships. Motivation Science. 2018 Mar;4(1):60–77.

4. Denzinger F, Backes S, Job V, Brandstätter V. Age and gender differences in implicit motives. Journal of Research in Personality. 2016 Dec;65:52–61.

5. Denzinger F, Brandstätter V. Stability of and Changes in Implicit Motives. A Narrative Review of Empirical Studies. Frontiers in Psychology [Internet]. 2018 May 25 [cited 2019 Mar 18];9. Available from: https://www.frontiersin.org/article/10.3389/fpsyg.2018.00777/full

6. Kuhn R, Bradbury TN, Nussbeck FW, Bodenmann G. The power of listening: Lending an ear to the partner during dyadic coping conversations. Journal of Family Psychology. 2018 Sep;32(6):762–72.

7. Kuhn R, Hilpert P, Bodenmann G. Dyadic Coping among Swiss Couples. In: Falconier MK, Randall AK, Bodenmann G, editors. Couples Coping with Stress: A Cross- Cultural Perspective. New York: Routledge; 2016. p. 86–104.

8. Kuster M, Backes S, Brandstätter V, Nussbeck FW, Bradbury TN, Sutter-Stickel D, et al. Approach-avoidance goals and relationship problems, communication of stress, and dyadic coping in couples. Motivation and Emotion. 2017 Oct;41(5):576–90.

9. Kuster M, Bernecker K, Backes S, Brandstätter V, Nussbeck FW, Bradbury TN, et al. Avoidance orientation and the escalation of negative communication in intimate relationships. Journal of Personality and Social Psychology. 2015;109(2):262–75.

10. Landis M, Bodenmann G, Bradbury TN, Brandstätter V, Peter-Wight M, Backes S, et al. Commitment and Dyadic Coping in Long-Term Relationships. GeroPsych. 2014 Jan;27(4):139–49.

11. Leuchtmann L, Milek A, Bernecker K, Nussbeck FW, Backes S, Martin M, et al. Temporal dynamics of couples’ communication behaviors in conflict discussions: A longitudinal analysis. Journal of Social and Personal Relationships. 2018 Oct 25;026540751880658.

12. Leuchtmann L, Zemp M, Milek A, Nussbeck FW, Brandstätter V, Bodenmann G. Role of clarity of other’s feelings for dyadic coping: Clarity of feelings and dyadic coping. Personal Relationships. 2018 Mar;25(1):38–49.

13. Neysari M, Bodenmann G, Mehl MR, Bernecker K, Nussbeck FW, Backes S, et al. Monitoring Pronouns in Conflicts: Temporal Dynamics of Verbal Communication in Couples Across the Lifespan. GeroPsych. 2016 Dec;29(4):201–13.

14. Rusu PP, Hilpert P, Turliuc MN, Bodenmann G. Dyadic Coping in an Eastern European Context: Validity and Measurement Invariance of the Romanian Version of Dyadic Coping Inventory. Measurement and Evaluation in Counseling and Development. 2016 Oct;49(4):274–85.

15. Zemp M, Backes S, Brandstätter V. The power motive and parenting style—Is incongruence related to inconsistency? Motivation Science. 2017 Dec;3(4):383–92.

16. Zemp M, Bodenmann G, Backes S, Sutter-Stickel D, Revenson TA. The Importance of Parents’ Dyadic Coping for Children: The Importance of Parents’ Dyadic Coping. Family Relations. 2016 Apr;65(2):275–86.

17. Zemp M, Bodenmann G, Backes S, Sutter-Stickel D, Bradbury TN. Positivity and Negativity in Interparental Conflict: Implications for Children. Swiss Journal of Psychology. 2016 Oct;75(4):167–73.

18. Zemp M, Nussbeck FW, Cummings EM, Bodenmann G. The Spillover of Child-Related Stress into Parents’ Relationship Mediated by Couple Communication: Impact of Child-Related Stress on Interparental Relations. Family Relations. 2017 Apr;66(2):317–30.

*Note*: Data presented in the present article are part of a large-scale study addressing different relationships variables. However, the current manuscript is the only one, which investigates stress, dyadic coping behavior and relationship satisfaction based on questionnaire data.
